# Animal Welfare and Mountain Products from Traditional Dairy Farms: How Do Consumers Perceive Complexity?

**DOI:** 10.3390/ani8110207

**Published:** 2018-11-14

**Authors:** Anna Zuliani, Lars Esbjerg, Klaus G. Grunert, Stefano Bovolenta

**Affiliations:** 1 Department of Agricultural, Food, Environmental and Animal Sciences, University of Udine, 33100 Udine, Italy; stefano.bovolenta@uniud.it; 2 MAPP Centre, Department of Management, Aarhus University, DK 8000 Aarhus, Denmark; lae@mgmt.au.dk (L.E.); klg@mgmt.au.dk (K.G.G.)

**Keywords:** mountain product, consumers’ perception, qualitative research, animal welfare, dairy cattle, small-scale farms

## Abstract

**Simple Summary:**

European consumers recognize the added value of mountain dairy production and relate it to a composite of positive attributes. However, while consumers’ understanding of conventional dairy production and animal welfare has already been investigated, how consumers perceive animal welfare in traditional mountain dairy farming remains unexplored. This qualitative study aims at shedding light on consumers’ perceptions regarding animal welfare in mountain dairy cheese production systems. Focus group interviews were carried out with three major consumer groups that purchase mountain cheese including rural consumers living in mountain villages, urban consumers living in the plains, and urban consumers living in mountain cities. The results of this study show that all participants expect mountain farming to be on a smaller scale and mountain products to be healthier when compared to non-mountain farming systems. However, the verbal appreciation of certain husbandry and management choices especially in the case of urban consumers did not result in their recognition when pictures of traditional husbandry systems were provided, which displays a disconnection between the expectations towards mountain production systems and reality. These findings will support the development of a transparent science-based dialogue among mountain dairy chain actors on animal welfare and sustainable farming practices in mountain areas.

**Abstract:**

This qualitative study aims to investigate consumers’ perceptions toward dairy cow welfare in traditional mountain farms. While consumers’ understanding of conventional dairy production and animal welfare has already been investigated, how consumers perceive animal welfare in traditional mountain dairy farming remains still unexplored. Focus group interviews were conducted with consumers having different degrees of geographical proximity to mountains and with an explicit interest in local dairy products. The results of this qualitative study show that participants expect mountain farming to be on a smaller scale when compared to non-mountain farming systems and expect mountain products to be healthier. Similarly, all participants consider origin, locality, and small-scale production as relevant quality attributes of mountain cheese. However, the appreciation of these abstract features did not necessarily result in their recognition when sample pictures of traditional husbandry systems were provided especially in the case of urban participants. This study contributes to reveal the gap between urban consumers’ conception of mountain farming and the actual farming practices. It also indicates the need to promote an effective science-based dialogue on animal welfare that goes beyond an anthropomorphic perspective and tackles the complexity of farming systems in relation to the context in which they are located.

## 1. Introduction

Mountain areas represent more than 40% of Europe’s landmass. Agriculture is part of the cultural and economic landscape and contributes significantly to the provision of ecosystem services and related public goods [1]. Mountain farms are generally small-scale because of the environmental and climate constraints that limit housing options and forage production. The resulting lower outputs, higher production costs, and the remoteness compared to lowland farms impair the economic competitiveness of this farming system and ultimately threatens its existence. In fact, in the Italian Alps alone, cattle farms decreased by more than 50% between 1990 and 2010 [1]. In spite of the crisis hitting small-scale and mountain producers, the majority of European consumers living in Alpine countries [2] recognize the added value of mountain production particularly cheese as the most important mountain product and relate it to a composite of positive attributes such as purity, authenticity, and simplicity.

Given the multiple positive externalities linked to mountain production systems as well as their fragility, the EU has introduced the optional quality term “mountain product” in the Regulation No. 1151/2012 and in the Delegated Act (EU) No. 665/2014 in an attempt to unlock the market potential of mountain products. The optional quality term provides consumers with information that these products originate in a mountain region, which is an example of credence quality attributes [3] since consumers have no ability to verify the accuracy of this type of product characteristics.

Animal welfare is another credence quality attribute [4] that is of great interest to consumers and could represent a further way to differentiate and support sustainable farming systems. Small-scale and extensive systems are perceived as inherently welfare-friendly [5]. Mountain dairy farms are generally small-scale (in Italy, 78% of mountain dairy farms are micro-scale enterprises with less than 20 cows) and provide pasture access during the summer. However, during the winter, animals are kept indoors and mainly tethered [6]. Despite several studies showing that good welfare outcomes are neither linked to farm-size [7] nor provision of resources [8], most citizens and consumers simply relate animal welfare to a single resource-based indicator, i.e., housing system. For example, the concern for housing conditions was expressed as an emphasis for values such as “freedom to move or fulfill natural desires” by Dutch consumers [9] or as a preference for pasture-based systems by British and German consumers [10,11]. Thus, cattle tethering may be considered as an unacceptable husbandry practice by most consumers even in specific climatic and topographic conditions such as those found in mountain farms.

As part of a larger project on mountain products and ecosystem services, this qualitative study aims at investigating consumers’ attitudes and perspectives towards dairy cow welfare in mountain farms. Three research objectives were identified in order to highlight potential communication gaps and opportunities between mountain producers and consumers with different degrees of proximity to mountain areas. The first objective was to understand the way consumers in three different contexts conceptualize the mountain environment and mountain farming. The second objective was to look into consumers’ knowledge and preferences for husbandry systems and animal welfare attributes to be used in mountain dairy farms. The third objective was to explore which quality attributes are expected by consumers in relation to mountain cheese.

## 2. Material and Methods

For this study, focus group discussions were chosen as the method to investigate consumers’ attitudes and perspectives on animal welfare in mountain farms and expected product quality attributes. Focus groups are a qualitative research technique deemed suitable to gather everyday knowledge on unexplored topics [12]. They can highlight underlying motivations and values, which are not generally revealed through closed interview questions. This approach has been used in market and social research since the 1950s in order to facilitate and bring forth a wide variety of viewpoints by the means of an active group interaction. Focus group discussions were carried out in adherence to the core principles of social research ethics. Verbal informed consent was obtained and recorded from all participants involved in the study.

### 2.1. Participant Information

Participants were recruited by using a purposive sampling method, according to different degrees of geographical proximity to mountains and with an explicit interest in local dairy products. This choice was driven by the assumption that this group of people might be the most motivated in purchasing “mountain products” and, at the same time, the most interested in additional process-related quality attributes such as welfare-friendly claims.

Three focus groups were conducted and involved 22 people in total (Table 1). Each focus group consisted of 6 to 9 people with the only criteria being that they were cheese purchasers/eaters belonging to a defined community.

According to Equation [13], the three main consumer groups that purchase mountain cheese in the eastern Alps such as rural consumers living in mountain villages of about 1000 inhabitants (rural mountain consumer, RMC), urban consumers living in the plains (plain urban consumer, PUC), and urban consumers living in mountain cities (mountain urban consumers, MUC) were identified. Both cities had about 100,000 inhabitants. The identification of these target groups also aimed at capturing the highest variability of structural determinants known to affect attitudes towards animal welfare [14] such as the urban-rural factor, socio-economic status, and previous animal-related experiences. Consumption and location profiles were deemed more important in explaining attitudes than age and sex and, thus, were the only inclusion/exclusion criteria used. Participants from RMC (*n* = 7) were recruited through project advertisement at the local cheese shop while PUC (*n* = 6) and MUC (*n* = 9) were recruited through organizations with an explicit interest in local food and environmental issues (e.g., Slow Food).

### 2.2. Focus Group Discussions

A moderator guided the discussions, which followed a semi-structured interviewing method. The discussion followed a funnel approach where general questions were asked first and more specific points on animal husbandry, animal welfare, and product quality attributes were addressed later in the discussion. At first, socio-demographic data were collected by using a written questionnaire. Before starting the discussion, a broad introduction on the EU Regulation concerning the “mountain product” was provided to the groups. Then participants introduced themselves to each other in terms of their job, hobbies, outdoor activities, and special dietary requirements.

The conceptualization of mountain farming was initially investigated by asking participants to make a collective drawing on what the idea of mountain/mountain environment was evoking from them and by sharing it with the others. Afterwards, participants were invited to write down and explain three words that best describe mountain farming compared to lowland farming. Pictures on six farming cases where different housing/management choices (i.e., tie-stall or loose housing, with and without pasture/paddock access, dairy or dual purpose breeds, horned or disbudded animals) for both summertime and wintertime were displayed and participants were asked to comment on them and later to rank them according to their preference.

While the main focus was put on the housing system, horned and dehorned cows as well as different breeds and farm sizes were also represented in the pictures. Subsequently, the indicators currently used to assess dairy cow welfare based on the EFSA protocol for small scale farms [15], which was adapted after the Welfare Quality framework [16] was provided to participants. They were invited to discuss the framework and eventually to rank the indicators according to their priority in terms of farm animal welfare. As a final step, consumers were asked to provide their opinion on the concept of quality when related to a cheese produced in mountain farms.

The group discussions were video-recorded, transcribed verbatim, and translated to English. All conversations were coded and analyzed in order to identify themes that emerged as being important for the participants and classified accordingly. Analysis was assisted by the use of Nvivo software (QSR International, Version 11, 2015, London, UK).

## 3. Results

### 3.1. Conceptualization of the Mountain Environment and Mountain Farming

Participants from PUC depicted the mountain environment mainly with stereotypical features such as mountain peaks with snow and summit crosses, a shepherd attending cows wearing bells, ski lifts, paths for bikes and people, a river, and meadows with flowers.

**Figure 1:**
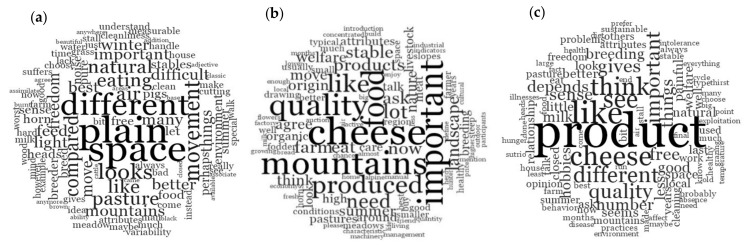
Conceptualization of mountain environment and mountain farming by: (**a**) plain urban consumers, PUC, (**b**) mountain urban consumers, MUC, and (**c**) rural mountain consumers, RMC.

When asked what the concept of the mountain was evoking from them, RMC participants drew objects related to their daily life such as houses, wild plants and mushrooms, trees for firewood, water streams, wildlife, and domesticated ruminants. While MUC drew similar features, they also mentioned the downsides of the mountain environment such as steep slopes, forest encroachment, and weather variability. The hardship of mountain farming as a job was highlighted by PUC only when asked to write down three words on mountain farming and was not pointed out at all by RMC. The concept of a healthy environment expressed as “quality” (Figure 1b,c) in comparison to other farming systems (“different,” Figure 1a,c) were the most mentioned characteristics of mountain livestock farming in all groups. RMC participants put plenty of emphasis on the fact that mountain farming by being small-scale is more sustainable and uses “a resource and does not exploit it until it is depleted” (RMC, female in her 30s). Moreover, due to being closer to the production site, they perceived that they could “see” (Figure 1c) and, thus, trust the entire supply chain. For both RMC and MUC, mountain farming ensures the maintenance of traditional productions as well as a traditional landscape “that is very similar to that of my grandfather” (RMC, male in his 40s). For MUC, mountain farms also have a role to play in biodiversity conservation, which means not only plant species but also rare cattle breeds. The concept of biodiversity was also postulated by PUC who mentioned that cows in mountain farms have more freedom to move and are surrounded by a noiseless environment.

### 3.2. Husbandry System Preference and Acceptance

**Figure 2:**
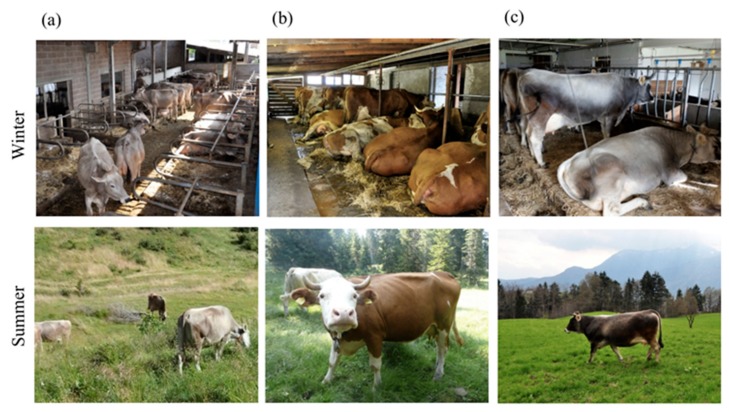

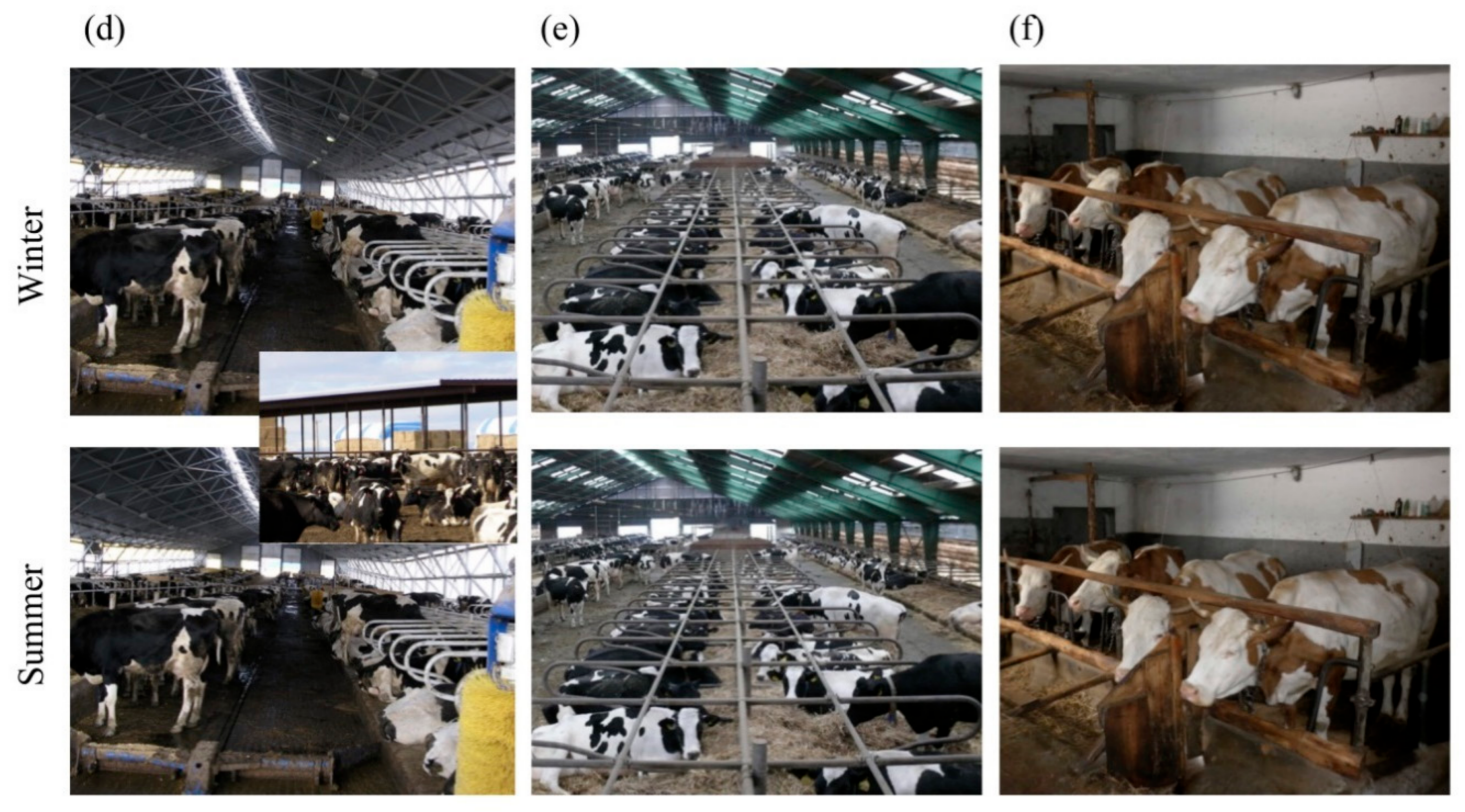
Pictures of six husbandry systems (i.e., cases (**a**–**f**)) representing a variety of housing, breed, and management choices during winter and summertime for dairy cattle.

When looking at the pictures of different husbandry systems (Figure 2), participants from RMC were struck by the different farm sizes and expressed their preference for small scale farms because “the cow is seen as an animal not as a milk dispenser” (RMC, female in her 30s) and “maybe they are brushed every day…. I expect this last farm (i.e., Figure 2f) not to be focused on profit, it is probably more a hobby than anything else” (RMC, male in his 60 s). One participant expressed strong concern “about the fact that having so many animals, the risk of epidemics and illness leads to the use of drugs and antibiotics, so the magnitude of product usage will be greater in case d and e” (RMC, female in her 20s). These concerns probably affected RMC’s choice when asked to rank their favorite (i.e., Figure 2b) and least favorite (i.e., Figure 2e) husbandry systems.

Stronger emphasis was put by urban consumers on pasture access and freedom of movement. They also referred to the importance of “light” (PUC, females in their 40s) and “fresh air” (PUC, female in her 30s). PUC were also concerned about cleanliness of the cows and manure management system. MUC pointed out the presence of horned cows and mentioned that “it is important that cows have horns because they need them for displaying their behavior in the herd” (MUC, female in her 40s). Both urban groups agreed on the most favored (i.e., Figure 2a) and least favorite (i.e., Figure 2f) husbandry system.

### 3.3. Welfare Indicators’ Preferences and Knowledge

When participants were asked to discuss the criteria that they used to choose the best and the worst farming systems and what welfare indicator they considered the most important, all agreed on the need for adequate space for the cows either in tie-stalls or loose housing systems. RMC again brought up the idea that “everything depends on the number of animals, fewer animals receive better treatment” (RMC, female in her 30s). In addition, they mentioned that cows looked happier and less lean in the farming systems they preferred.

For MUC and PUC, the quality of feed as well as the relationship the farmer has with the cows contribute to animals’ well-being.

Despite the participants raising similar themes, the ranking exercise on the indicators currently used to assess welfare on small-scale farms revealed some differences in the priorities the different groups hold (Table 2). When consumers were asked to rank welfare attributes currently used to carry out the assessments of cows’ welfare in mountain farms, urban consumers attributed great importance to good feeding principles. MUC expressed strong concern regarding painful practices such as dehorning and disbudding in contrast to PUC who considered the practice of little concern and actually they were not fully aware of it (“is it a common practice?” and “do animals suffer?” PUC, females in their 40s). For RMC, the absence of disease was the most important indicator of well-being. In terms of least important indicators, all groups mentioned that they ranked natural behavior as the least important indicator not because they considered it not important but because they felt it was going to be achieved if all others criteria were met.

### 3.4. Cheese Quality Attributes

When participants were asked to discuss what contributed to quality in relation to mountain cheese, all agreed that origin, local production, and longer ripening time were important characteristics. MUC emphasized their interest for cheese produced with “high quality hay milk” (MUC, male in his 50s) and coming from organic, extensive, and small-scale production systems. This latter aspect was seen as a synonym of quality also by PUC. Additionally, PUC were extremely interested in the sensory attributes of cheese such as smell, color, and flavor.

## 4. Discussion

While the level of public understanding of dairy production and animal welfare has already been investigated [17,18,19], the attitudes of consumers toward animal welfare in mountain farming systems is still unexplored. Given the optional quality term “mountain product” that was introduced in the EU and progressively transposed by member countries into the national legislation as well as the growing consumers concern toward animal welfare, we undertook this study intended to elicit consumers’ views and to shed light on the previously mentioned topics.

Three focus group interviews with consumers with different degrees of geographical proximity to mountains and with an explicit interest in local dairy products were conducted. In agreement with previous findings [20] and despite background differences among participants, all groups conceptualized mountain areas as a healthy environment where healthy fodder and healthy animals produce healthy food. Mountain farming was also expected to be small-scale and was acknowledged to use sustainable local resources and preserve biodiversity. Similar results were also reported in Reference [21] when urban consumers were asked to rank mountain ecosystem services. In fact, the most mentioned concepts were landscape aesthetics, biodiversity maintenance, and food quality.

In terms of husbandry systems’ preference, all groups believed that access to mountain pasture during the summer time was an essential aspect for ensuring animal well-being. However, there were different views over the winter husbandry system. Urban consumers preferred loose housing systems and free-range systems over tie-stall systems and no acceptance was shown for permanent tethering, which is similar to what Reference [10] reported. In contrast, mountain consumers expressed no concern for tie-stall systems because of the tight relationship they perceive between small-scale and low burden of disease as well as between low burden of disease and animal well-being.

However, when looking at the ranking exercise on welfare indicators currently used to assess animal welfare in small-scale farms, it appears that consumers hold contrasting views simultaneously. Good housing and natural behavior principles were not considered a priority in comparison to good feeding and good health principles, which is similar to what was reported by Reference [22] in a similar exercise with Flemish farmers and consumers. In addition, a note has to be made on the attitude toward disbudding/dehorning, which has been investigated among farmers [23], but it is almost unexplored among consumers. In this study, participants displayed a wide range of knowledge and concern about this practice. In fact, in contrast to PUC who were unaware about the practice, MUC showed concern about the potential issues related to the practice of disbudding/dehorning and coherently set the absence of painful practices, which includes disbudding/dehorning, as a priority in ensuring animal well-being. However, when asked to choose a favorite farming system, participants from both PUC and MUC picked a picture of a farm (Figure 2a) in which all cows underwent disbudding procedures. Additionally, while small scale was considered a distinctive feature of mountain farms and a key attribute of quality for both MUC and PUC, this belief was not followed up by the choice of a small-scale farm in the husbandry systems exercise, which displays a “realistically informed idealism” [19] or a “functional ignorance” [9] towards mountain farming. In such attitudes, consumers theoretically recognize limits and added values of certain farming systems but, in practice, they struggle in acknowledging its real features. For what concerns mountain cheese quality attributes and animal welfare information, focus group participants associated cheese quality mostly to credence attributes such as origin and sensory attributes such as ripening time. In contrast to what was reported in Reference [24], none of the participants mentioned safety and healthiness as important quality attributes possibly because of origin especially in the case of mountain cheese, which is associated with trustworthiness and authenticity of production. The only association that participants made between cheese quality attributes and animal welfare was about the practice of summer pasturing and its effect on desired cheese sensory properties. While it has been shown that the provision of selected information on process-related attributes such as summer husbandry system increases the expected liking of mountain cheese [25], it seems that additional information (e.g., painless practices or enhanced human-animal relationship) would require good knowledge of livestock farming in order to be assimilated and appreciated despite their importance in current welfare assessment protocols [26].

It is worth noticing that the results presented here might have been affected by the priority given to the rural/urban factor as the main inclusion criteria for taking part of the study. It is known that other structural determinants such as gender, age, income, education, and personal experiences with farming [14] may contribute to the development of attitudes towards animal welfare. This study confirms that participants living in rural areas have a more positive perception of animal welfare in traditional mountain farming systems. However, it is important to highlight that, while gender was evenly distributed among groups, participants in RMC were generally younger, less educated, and have lower incomes when compared with urban participants. These factors, which are linked to fewer concerns toward animal welfare [14], may have further influenced our results and widened the gap between urban and rural perceptions.

## 5. Conclusions

The results of this qualitative study show that consumers expect mountain farming to be small scale and mountain products to be healthier than low-land products. However, while participants from the small mountain community link these features to farming systems highly representative of the mountain areas in terms of both breeds and the farm scale and husbandry system, urban consumers struggled to acknowledge the real features of mountain farming systems.

Insights into consumers’ perceptions and attitudes are necessary to involve all stakeholders in the ongoing debate on animal welfare especially in fragile systems such as those located in mountain areas. This study contributes to reveal the gap between urban consumers’ conception of mountain farming and the actual farming practices. It also indicates the need to promote an effective science-based dialogue on animal welfare that goes beyond an anthropomorphic perspective and tackles the complexity of farming systems in relation to the context in which they are located. Specific actions may include the implementation of dissemination events targeting different consumer groups and the development of labeling scheme for mountain products with additional information on valuable mountain farming practices.

## Figures and Tables

**Table 1:** Socio-economic characteristics and cheese consumption habits of plain urban consumers (PUC), mountain urban consumers (MUC), and rural mountain consumers (RMC).

Variables	Classes	PUC, *n* = 6	MUC, *n* = 9	RMC, *n* = 7
Age (years)	18–30	0	2	3
	31–45	3	3	2
	46–60	2	4	0
	>60	1	0	2
Gender	Female	3	5	3
	Male	3	4	4
Education	Secondary	4	3	5
	Graduate	2	6	2
Income (€)	<30,000	0	1	5
	31,000–45,000	1	5	2
	46,000–60,000	3	2	0
	>61,000	2	1	0
Dairy Farm Visits	Never	1	0	2
	Once	2	1	3
	More than 5 times	3	8	2
Cheese Consumption	Daily	1	3	2
	2–4 times/week	3	5	4
	Once a week	2	1	1

**Table 2:** Preferences (1 = most important, 5 = least important) on dairy cattle welfare attributes as expressed by plain urban consumers (PUC), mountain urban consumers (MUC), and rural mountain consumers (RMC).

Principles	Criteria	PUC, *n* = 6	MUC, *n* = 9	RMC, *n* = 7
Good feeding	Absence of hunger	1	1	2
	Absence of thirst	1	1	2
Good housing	Animal cleanliness	4	4	3
	Loose housing system	2	2	3
Good health	Absence of injuries	3	4	3
	Absence of diseases	2	3	1
	Absence of pain (disbudding/dehorning)	5	1	3
Natural behavior	Good human-animal relationship	3	3	4
	Appropriate behavior	5	5	5
